# The effectiveness of high molecular weight hyaluronic acid for knee osteoarthritis in patients in the working age: a randomised controlled trial

**DOI:** 10.1186/s12891-019-2546-8

**Published:** 2019-05-07

**Authors:** Job Hermans, Sita M. A. Bierma-Zeinstra, Pieter K. Bos, Dieu Donne Niesten, Jan A. N. Verhaar, Max Reijman

**Affiliations:** 1 Department of Orthopaedic Surgery, Erasmus University Medical Centre Rotterdam, PO Box 2040, 3000 CA Rotterdam, The Netherlands; 2 Department of General Practice, Erasmus University Medical Centre, PO Box 2040, 3000 CA Rotterdam, The Netherlands; 3 Department of Orthopaedic Surgery, Reinier de Graaf Hospital, PO Box 5011 2600, GA Delft, The Netherlands

**Keywords:** Knee osteoarthritis, Hyaluronic acid, Effectiveness, Randomised controlled trial, Working age

## Abstract

**Background:**

High molecular weight (HMW) hyaluronic acid (HA) is a treatment option for knee osteoarthritis (OA). The efficacy of HMW-HA in knee OA is investigated extensively, but the effectiveness in patients in the working age is unknown. Nevertheless, the number knee OA patients in the working age is increasing. Surgical treatment options are less eligible in these patients and productivity losses are high. In this study the effectiveness of intra-articular HMW-HA added to regular non-surgical usual care in everyday clinical practice (UC) compared to UC over 52 weeks in symptomatic knee OA patients in the working age was investigated.

**Methods:**

In this open labelled randomized controlled trial, subjects aged between 18 and 65 years with symptomatic knee OA (Kellgren and Lawrence I-III) were enrolled and randomized to UC + 3 weekly injections with HMW-HA (intervention) or UC only (control). The primary outcome was the between group difference in responders to therapy according to OMERACT-OARSI criteria after 52 weeks. These criteria include the domains pain, knee related function and patient’s global assessment (PGA). Function was evaluated with the KOOS questionnaire. Pain was assessed with the Numeric Rating Scale. Secondary outcome comprised the between group difference on the individual responder domains, as analysed with a random effects model. Odds Ratios (OR) were calculated by logistic regression analysis. Sensitivity analyses were performed.

**Results:**

In total, 156 subjects were included (intervention group 77, control group 79). Subjects in the intervention group (HMW-HA + UC) were more often responder compared to the controls (UC). Depending on whether pain during rest or pain during activity was included in the responder domains, 57.1% versus 34.2% (*p* = 0.006) and 54.5% versus 34.2% (*p* = 0.015) was responder to therapy respectively. The results of the secondary outcome analyses show that scores on individual responder domains over all follow-up moments were statistically significant in favour of the intervention group in the domains pain during rest (δ 0.8, 95%CI 0.2; 1.4, *p* = 0.010), knee related function (δ − 6.8, 95%CI -11.9; − 1.7, p = 0.010) and PGA (δ − 0.7, 95%CI -0.9; − 0.4, *p* < 0.0001).

**Conclusions:**

Intra-articular HMW-HA added to usual care is effective for knee OA in patients in the working age.

**Trial registration:**

www.trialregister.nl, NTR1651, registered 2009-3-3.

**Electronic supplementary material:**

The online version of this article (10.1186/s12891-019-2546-8) contains supplementary material, which is available to authorized users.

## Background

Knee osteoarthritis (OA) is a chronic degenerative disease of the knee joint, causing pain, joint stiffness and functional impairment [1–3]. The lifetime risk on symptomatic knee OA is over 40% [4]. Next to health impairment and disability, knee OA is associated with substantial healthcare consumption and costs [1, 5, 6].

The initial pharmacological treatment for patients with symptomatic knee OA generally includes rapid-acting pain medication like acetaminophen or non-steroidal anti-inflammatory drugs (NSAIDs). NSAIDs have shown to be effective in pain reduction and functional improvement in the symptomatic treatment of knee OA [7–9].

Treatment with NSAIDs is related to an increased risk of serious gastrointestinal and cardiovascular side effects, indicating limited use of NSAIDs only [10, 11]. The safety profile of NSAIDs contradicts with the chronic character of knee OA in which prolonged symptomatic treatment is often required. Additionally, non-pharmacological interventions such as strength training, exercise and weight management are added to the treatment regime [12–14].

An alternative treatment for knee OA patients is intra-articular injection therapy with hyaluronic acid (HA) [15]. Intra-articular HA results in similar effects on pain reduction and improvement of function compared to NSAID use, without the aforementioned side effects [8, 16, 17]. The efficacy of intra-articular HA has been investigated extensively in randomized controlled trials (RCTs) and subsequently in various systematic reviews and meta-analyses [12, 15]. Peak effectiveness of a series of intra-articular HA is reached between 1 and 2 months and residual effects exist up to 6 months [15, 16, 18].

Limiting the results of meta-analyses to high quality trials only, the effect on pain is still clinically relevant in favour of intra-articular HA [14, 18]. There is increasing evidence that within the spectrum of available HA derivatives the efficacy of HA products with a high molecular weight (HMW) is superior to the efficacy of derivatives with a low molecular weight [19, 20].

The effectiveness of HMW-HA in knee OA patients in the working age has not been evaluated yet. Relevance lies in the fact that the number patients with knee OA in the working age is increasing and surgical treatment options like unicompartmental or total knee arthroplasty (TKA) are less eligible in these patients, especially when they are involved in a physically demanding occupation [21, 22]. The revision rate of knee arthroplasty in these patients is high and the life span of the prosthesis is limited [23]. Furthermore, the costs from loss of productivity at work due to symptomatic knee OA are high in patients in the working age [24]. In this population, the availability of an effective local therapy in everyday clinical care could thus offer important healthcare benefits next to possible economic benefits.

The aim of this study was to assess the effectiveness of intra-articular HMW-HA added to usual care (UC) compared to UC over a period of 52 weeks in symptomatic knee OA patients in the working age. We hypothesized that adding HMW-HA in patients with knee OA has a clinical relevant effect.

Alongside this effectiveness analysis, a parallel economic evaluation was performed which was published previously [25]. In this article we report that adding HMW-HA to the usual care results in an increase in quality of life. The increase is accompanied with an increase in costs. Ultimately this leads to a cost-effectiveness ratio of €9.100/ quality adjusted life years (QALY). Given the maximum willingness to pay for similar conditions to knee OA we conclude that intra-articular HMW-HA added to usual care for knee OA is probably cost-effective in the treatment of knee OA.

## Methods

The current effectiveness evaluation and the previously published cost-effectiveness evaluation are both part of the VIScosupplementation for Knee osteoarthritis (VISK) study. The VISK study is registered at the Dutch trial register (www.trialregister.nl, NTR1651). The study protocol is available from the corresponding author on request.

The VISK study does not include a placebo group. In light of the evidence on the efficacy of HMW-HA in knee OA, we specifically sought to investigate the actual effectiveness of this intervention. Such a study design, in which the intervention is compared to what is considered regular care that is provided in an everyday clinical setting (without a placebo), is required to facilitate the parallel economic evaluation of the VISK study [26, 27].

### Study sample

Inclusion of eligible subjects took place between May 2009 and May 2010 in 2 hospitals (1 academic, 1 non-academic) in The Netherlands. Consecutive knee OA patients at the outpatient orthopaedic department meeting the inclusion criteria were considered eligible. Patient’s age was set between 18 and 65 years, the latter being the pensionable age in The Netherlands at the inclusion period. Inclusion criteria were: pain > 3 months, mean pain severity ≥2 on the numeric rating scale (NRS), Kellgren & Lawrence (K&L) grade I to III in medial and/or lateral compartment.

Exclusion criteria were: intra-articular HA injections < 1 year, intra-articular steroid injection < 3 months, arthroscopy < 6 months, tibial osteotomy < 1 year, synovectomy, scheduled knee surgery < 1 year, varus/valgus deformity > 12 degrees, chondrocalcinosis, dermatologic knee disorders, allergy to HMW-HA components, (planned) pregnancy or lactation, inflammatory arthritis, severe hip OA, non-knee related regular analgesic use, daily oral steroid therapy, poor general health, conditions interfering with functional assessments, alcoholism, patients unable to attend follow-up and insufficient command of the Dutch language.

### Sample size, randomization and masking

The sample size was calculated to detect a between group difference of 20% in the primary outcome parameter which was defined as response to therapy at 52 weeks according to OMERACT-OARSI criteria [28]. A power of 80% and an alpha of 0.05 resulted in a required sample size of 64 subjects per group (128 subjects in total). Anticipating a 20% dropout over 52 weeks, the final required sample size was set at 154 subjects.

Randomization took place after informed consent was signed. Concealed randomization was performed by computer generated lists with randomly assigned blocs of 2, 4, 6, 8 or 10 subjects. An independent employee not involved in any other part of the study performed the randomization. Stratification took place for radiologic degree of knee AO (K&L grade I/II versus grade III) and per orthopedic surgeons responsible for injections (2 per hospital, 4 in total).

The statistician and investigator responsible for assessment and analyses of the data were blinded for the treatment allocation. Due to the study design included subjects and orthopedic surgeons administering the study intervention could not be blinded.

### Interventions

Subjects in the intervention group received 3 weekly intra-articular injections with Hylan G-F 20 (Sanofi S. A, Paris, France) added to usual care or usual care only. Hylan G-F 20 is the HMW-HA derivative with the highest molecular weight available for clinical use (6000 kDa. The injections were performed through the superolateral approach [29]. Usual care was defined accordingly to the guidelines on the treatment of knee OA of the Dutch Orthopedic Association. This guideline recommends several non-surgical treatment modalities including pain medication (eg acetaminophen or NSAIDs), physical therapy and lifestyle recommendations [12]. Treating physicians were encouraged to follow these guidelines, but no treatment restraints were imposed. Other treatments were allowed when deemed appropriate in order to maintain the pragmatic character of the trial.

### Questionnaires

The follow-up was 52 weeks and data was collected through questionnaires by mail at baseline, 6, 13, 26, 39 and 52 weeks. Knee related function was assessed by the functioning in daily living scale of the Knee injury and Osteoarthritis Outcome Score (KOOS) [30, 31]. A normalized score from 0 (extreme symptoms) to 100 (no symptoms) was calculated for this subscale. Pain during rest and pain during activity was evaluated by the NRS, resulting in a score between 0 (no pain) and 10 (most severe pain) [32]. Patient’s global assessment (PGA) was assessed on a 5-point Likert scale on which subjects indicate the amount of improvement of their knee complaints compared to baseline (1. fair improvement, 2. moderate improvement, 3. no change, 4. moderate deterioration, 5. fair deterioration). Medication use and patient reported adverse events were monitored at all follow-up moments.

### Outcomes

The primary outcome was defined as response to therapy at 52 weeks follow-up according to OMERACT-OARSI criteria. This variable presents the results of changes after treatment in three symptomatic domains (pain, function, and PGA) as a single variable [28]. Response to therapy according to the OMERACT-OARSI criteria is defined as ≥10% absolute improvement and ≥ 20% overall improvement at final follow-up in at least 2 of the 3 responder domains (pain, function and/or PGA); or ≥ 20% absolute improvement and ≥ 50% overall improvement in either the pain or function domain.

The secondary outcome comprised the between group difference over the whole follow-up period of the 3 individual primary outcome responder domains: pain, function, and PGA.

### Statistical analyses

For the primary outcome, the difference in percentage of responders according to OMERACT-OARSI criteria between study groups after 52 weeks follow-up was calculated [28]. In the base case analyses two responder sets were investigated: 1. with pain during rest was included in the responder domains, next to function and PGA; and 2. with pain during activity included.

In order to minimize bias in favor of the intervention group, drop-outs and subjects lost to follow up were (regardless of their study results) considered non-responders in the intervention group, and (vice versa) responders in the control group in the final analyses.

Logistic regression analysis with responder as dependent variable and the intervention as independent variable were performed to calculate odds ratios (OR) including 95% confidence intervals (95%CI) after 52 weeks follow-up. The number needed to treat (NNT) to attain 1 responder was calculated (PASW statistics 17.0).

For the secondary outcome, scores on individual responder domains (pain during rest, pain during activity, knee related functioning in daily life, PGA) were analyzed over all follow-up moments by means of a random effects model with random intercept and slope. The baseline values of the variables and the treatment group were included in the model. In this way we obtained for each outcome an estimate for the between group difference in score on the relevant questionnaires (KOOS, NRS, Likert scale) over the whole follow-up period, including associated 95% CI (SAS 9.2, SAS Company).

Sixteen subjects divided over both study groups received knee related surgery during follow-up. This number was not foreseen and we therefore performed 2 additional sensitivity analyses to assess possible beneficial clinical effects on pain and function as a result of the surgery. These analyses were not specified in the VISK study protocol a priori. In these sensitivity analyses, subject receiving knee related were considered non-responder irrespective of their study results. [33] In the first additional analysis, subjects who received major knee related surgery (e.g. knee prosthesis implantation, high tibial osteotomy) during follow-up were considered non-responders. In the second additional analysis subjects receiving any knee surgery (major knee surgery plus minor knee surgery like arthroscopy or knee manipulation under general anesthesia) were considered non-responders. (PASW statistics 17.0) All analyses were performed according to the intention to treat principle. In order to generate unbiased estimates of the difference in effectiveness parameters across both treatment groups, we adjusted for baseline imbalances in and, if necessary, for unbalanced covariates.

## Results

### Study population

**Fig. 1:**
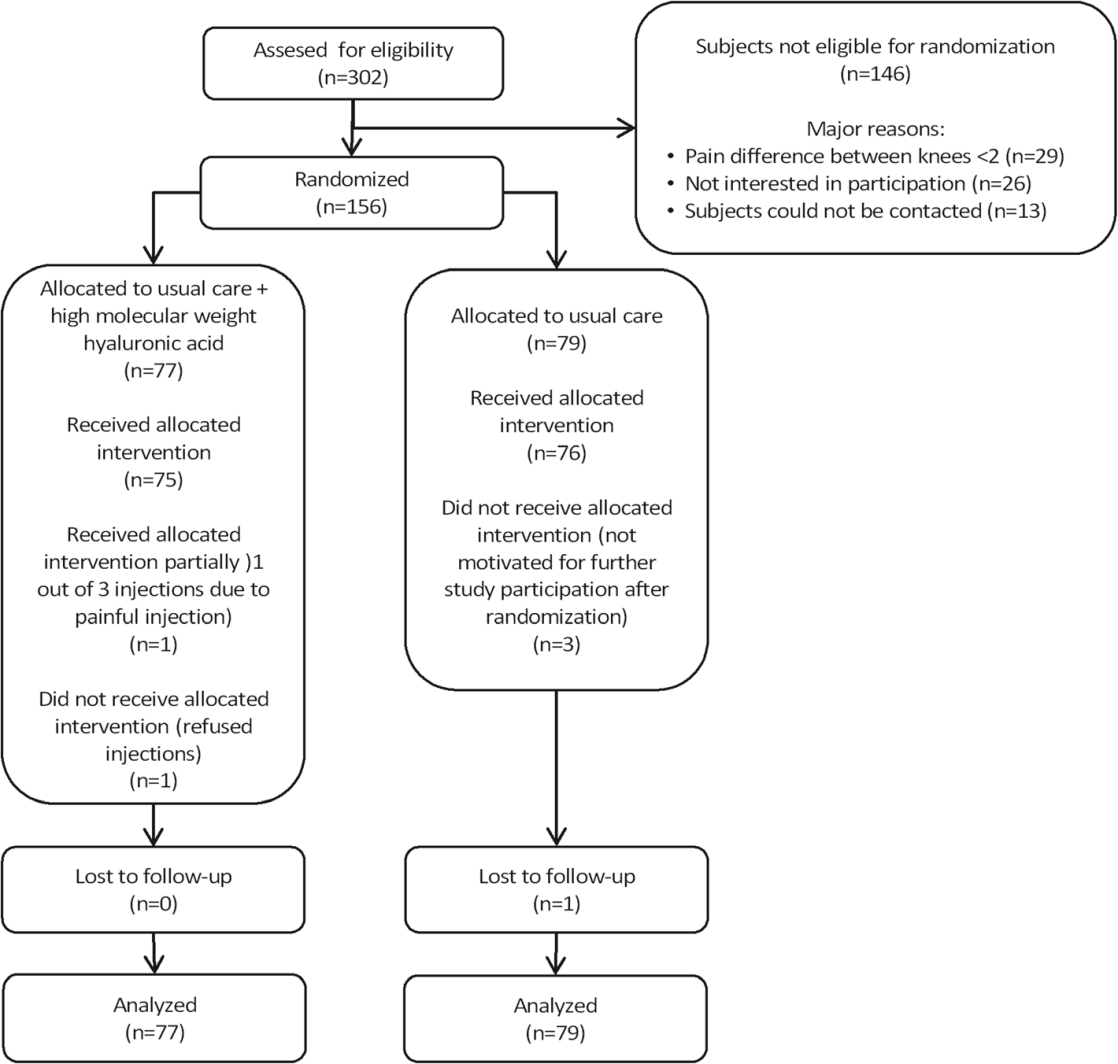
Flowchart

In total, 156 patients were included of which 77 subjects (mean age 53.6, standard deviation (SD) 8.6 range 20.9–64.6) in the intervention group and 79 subjects (mean age 54.8, SD 6.4, range 32.9–64.9) in the control group. The study flowchart is shown in Fig. 1. Additional characteristics of included subjects are shown in Table 1. One subject in the intervention group received only 1 out of 3 planned injections with HA due to a painful first injection and 1 subject refused the injections of HMW-HA after allocation to the intervention group. In the control group, 3 subjects were not motivated for further study participation after baseline measurements and randomization, and 1 subject was lost to follow-up. All subjects were retained in the analyses of their randomization groups. We adjusted for the baseline imbalances on pain and functioning in all analyses.

**Table 1:** Population characteristics (*n* = 156)

	intervention (*n* = 77)	control (*n* = 79)
mean age, years (sd, range)	53.6 (8.6, 20.9–64.6)	54.8 (6,4, 32.9–64.9)
female, *n* (%)	37 (48)	40 (51)
BMI, kg/m^2^ mean (sd, range)	28.9 (5.2, 20.4–44.8)	29.2 (5.4,19.4–43.5)
K&L I-II, *n* (%)	44 (57)	47 (59)
K&L III, *n* (%)	33 (43)	32 (41)
duration knee complaints 3-12 M, n (%)	43 (56)	36 (46)
duration knee complaints > 12 M, n (%)	34 (44)	43 (54)
pain during rest (0–10) ^1^, mean (sd, range)	4.8 (2.5, 0–8.0)	4.1 (2.6, 0–10)
pain during activity (0–10) ^1^, mean (sd, range)	6.5 (2.4, 0–10)	5.8 (2.4, 0–10)
quality of life (0–1)^2^, mean (sd, range)	0.68 (0.23, −0.05-1)	0.71 (0.24, −0.11-1)
KOOS subscales (0–100), mean (sd, range)		
*pain*	46.6 (20.6, 5.6–100)	52.5 (21.1, 11.1–100)
*other symptoms*	55.7 (18.3, 17.9–100)	61.3 (21.8, 3.6–100)
*function in daily life*	53.2 (20.2, 7.4–100)	60.2 (24.0, 10.3-100)
*function in sports & recreation*	24.0 (25.7, 0–95.0)	31.1 (30.9, 0–100)
*knee related quality of life*	30.8 (18.5, 0–68.8)	35.9 (18.7, 0–81.3)

^1^on Numeric Rating Scale, ^2^on EQ-5D questionnaire, K&L: Kellgren&Lawrence scale

### Primary outcome

In Table 2 the results on the primary outcome and the results of the sensitivity analyses are shown. Subjects in the intervention group were statistically significant more often responder to treatment arm they were randomized to compared to the control group. When pain during rest was included in the responder domains, 57.1% of the subjects in the intervention group were responder to therapy, against 34.2% in the control group (*p* = 0.006). With pain during activity included, 54.5% of the subjects was responders to therapy in the intervention group versus 34.2% of the controls (*p* = 0.015).

**Table 2:** Percentage responders at 52 weeks follow-up (*n* = 156)

	intervention (*n* = 77)	control (*n* = 79)	NNT	OR (95% CI)	*p*
all subjects analysed					
responder set 1^a^	57.1%	34.2%	4.4	2.6 (1.3; 4.9)	0.006
responder set 2^b^	54.5%	34.2%	4.9	2.3 (1.2; 4.4)	0.015
1st additional analysis^c^					
responder set 1^a^	50.6%	31.6%	5.3	2.2 (1.2; 4.3)	0.022
responder set 2^b^	48.1%	32.9%	6.6	1.9 (1.0; 3.6)	0.072
2nd additional analysis^d^					
responder set 1^a^	50.6%	31.6%	5.3	2.2 (1.2; 4.3)	0.022
responder set 2^b^	48.1%	31.6%	6.1	2.0 (1.0; 3.8)	0.049

^a^pain during rest included in responder domains next to function and PGA, ^b^pain during activity included in responder domains next to function and PGA, ^c^subjects receiving major knee related surgery considered non-responder, ^d^Subjects receiving any knee related surgery considered non-responder, OR: Odds ratio, CI: confidence interval, NNT: number needed to treat

### Secondary outcome

**Fig. 2:**
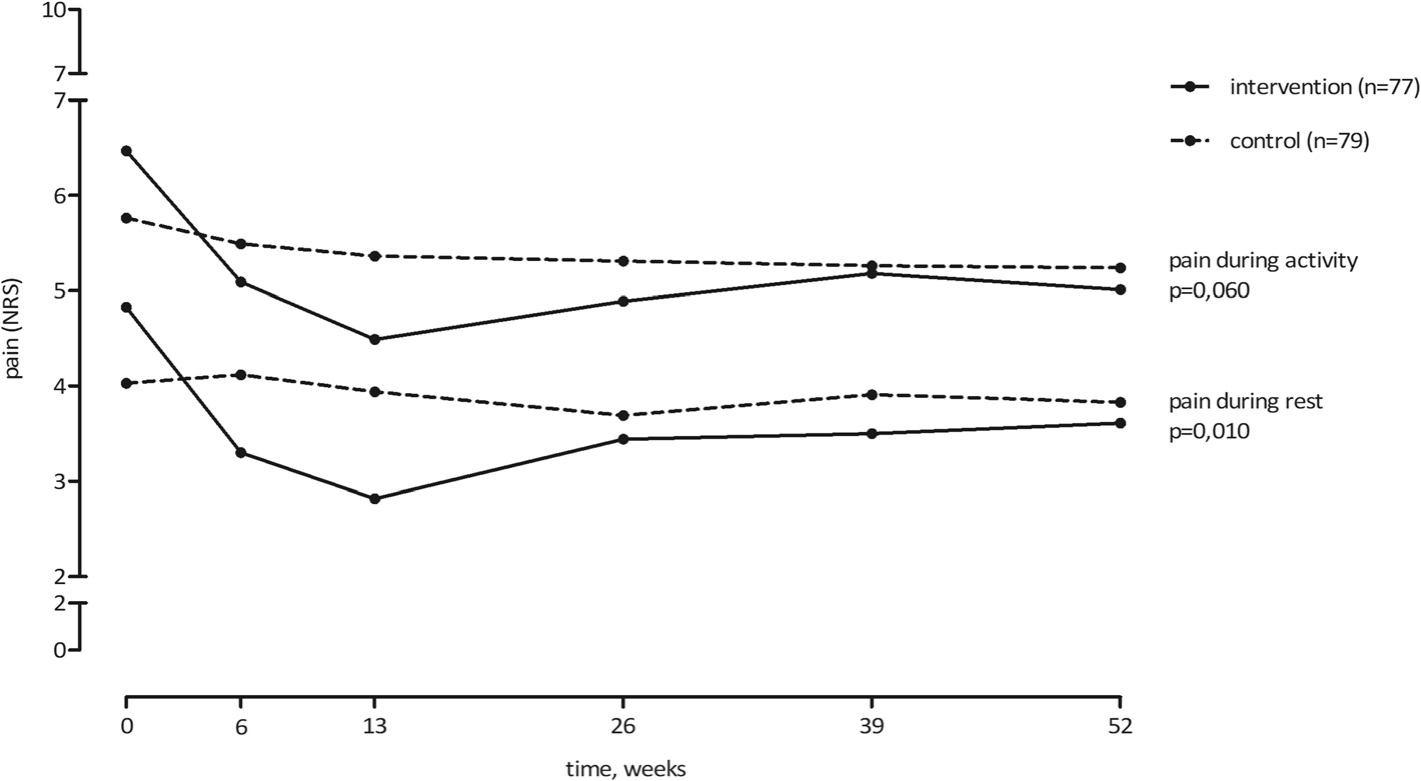
Results pain during rest and during activity

**Fig. 3:**
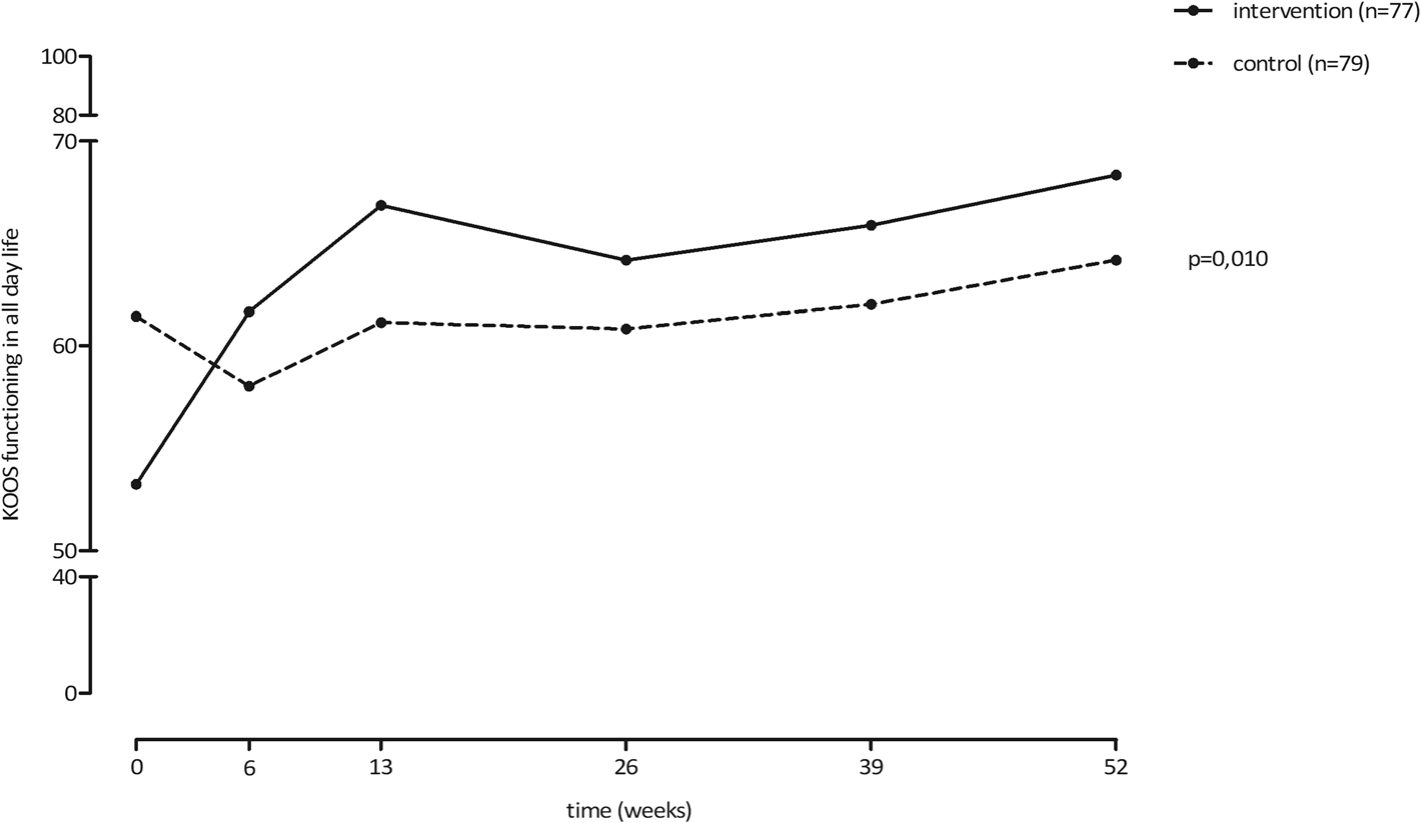
Results knee function

**Fig. 4:**
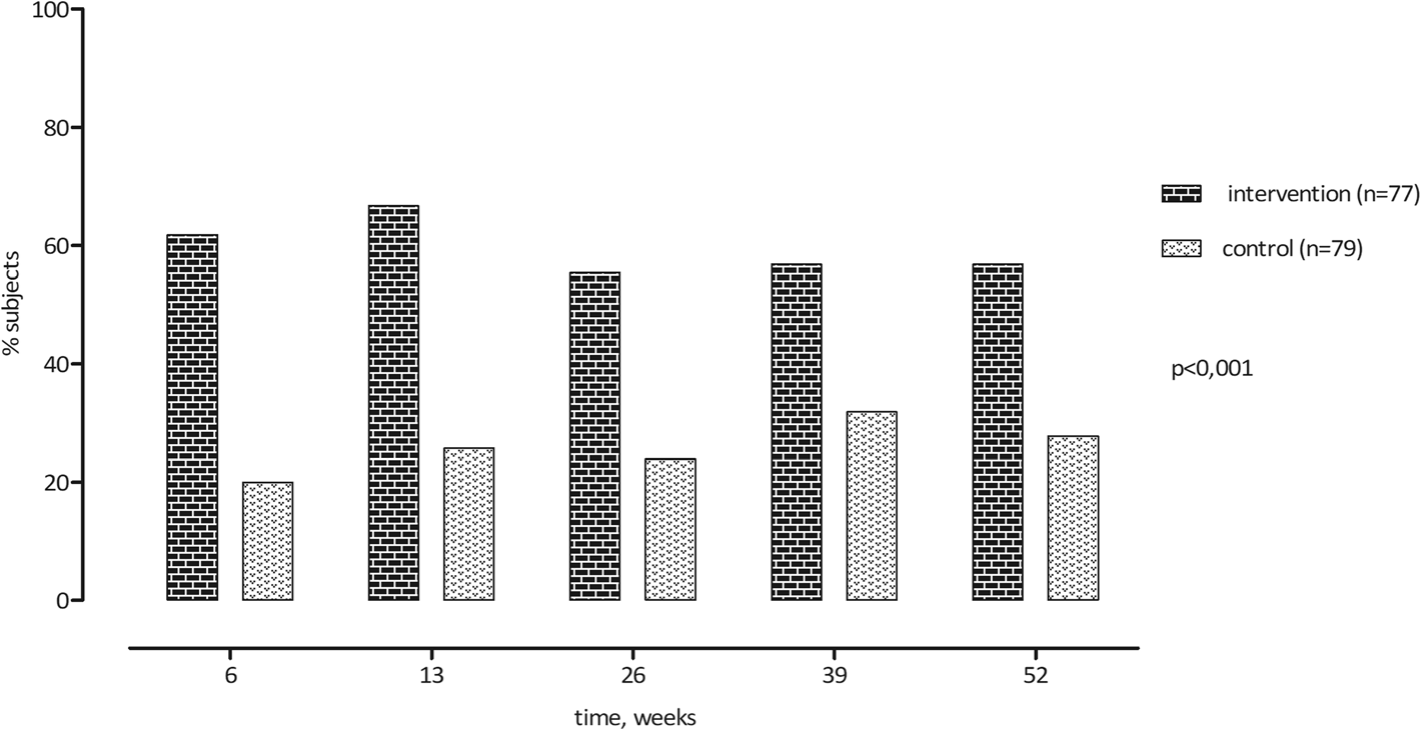
Results patients’ global assessment

Over the whole follow-up period, we found statistically significant better scores in the intervention group in the domains pain during rest, knee related function, and PGA (Figs. 2, 3 and 4). These results where statistically significant for pain during rest (δ 0.8, 95%CI 0.2; 1.4, *p* = 0.010), knee related function (δ − 6.8, 95%CI -11.9; − 1.7, *p* = 0.010) and PGA (δ − 0.7, 95%CI -0.9; − 0.4, *p* < 0.0001). The intervention group also scored lower on the pain during activity score, but this difference was not statistically significant (δ 0.6, 95%CI 0; 1.2, *p* = 0.060).

### Sensitivity analyses

Nine surgical procedures related to the study knee were performed in the intervention group during follow-up, versus 7 in the control group. Despite a slight decrease in the between group differences in responder percentages, the results of both additional analyses are still statistically significant in favour of the intervention group (Table 2).

### Medication use

At baseline, more subjects used pain medication because of knee complaints in the intervention group with (53%) compared to the control group (42%). This difference decreased over time, resulting in similar usage of pain medication for both groups at final follow-up. The difference in pain medication users was not statistically significant on any of the time points during follow-up.

### Adverse events

In the intervention group, more subjects reported any knee treatment related adverse events (AE) at 6 weeks (45% versus 18%. This difference was mainly due to flares or flare like symptoms of the study knee in this period (36% versus 10%), *p* > 0.001, number needed to harm (NNH) 4.0). The difference decreased at 13 weeks, and at 26 weeks the percentage of subjects reporting flares was similar in both groups. None of the between group differences on the following time points after 6 weeks were statistically significant. No septic arthritis of the study knee occurred in any of the subjects during follow-up.

The amount of non-treatment related AEs was similar in both groups during follow-up. An additional file shows the percentage of patients experiencing treatment and non-treatment related adverse events per study group per time point during study follow-up (see Additional file 1).

## Discussion

This study is the first to investigate the effectiveness of HMW-HA added to usual care in subjects with clinical knee OA in the working age. We showed that adding intra-articular injections with a HMW-HA derivative to usual care treatment in an everyday clinical setting resulted in statistically significant more responders to therapy. It resulted in improvement of pain, function and PGA in these patients. The between group difference on 3 out of 4 of the individual responder domains (pain during rest, knee related function, PGA) was statistically significant and in favour of the intervention group. Subjects in the intervention group experienced more episodes of transient knee pain and/or swelling during the first 6 weeks.

In our study we specifically choose to include subjects in the working age (mean age 54) with a higher involvement in paid work (75%) [25]. By doing so we were able to investigate the effectiveness of HMW-HA in a population in which knee OA levels are rising and in which surgical treatments like arthroplasty are less eligible due to high revision rate and limited life span of the prosthesis [21–23]. We showed that in this population, intra-articular HMW-HA leads to clinically relevant improvement in pain, function and PGA. Since the costs from loss of productivity at work due to knee OA are high in patients in the working age, the treatment with HMW-HA could also result in certain economic benefits [24]. This was investigated in the parallel economic evaluation of the VISK study, in which we report that intra-articular HMW-HA in knee OA is probably cost-effective in this population [25].

To date, 2 other studies compared HMW-HA added to usual care to usual care only [34, 35]. The same HMW-HA derivative as in our study was investigated. Both studies imposed no limitation on maximum age at time of inclusion, which probably contributed to the relatively low proportion of subjects involved in a paid occupation (19 to 34%) [34, 35]. In the first study statistically significant more patients in the intervention group were responder to therapy at final follow-up of 9 months [35]. The percentage of responders was higher in both study groups compared to our study. Also no restriction on the radiologic degree of OA was imposed in this study and the minimal pain score at entry was higher (4 against 2) [35]. The inclusion of clinically more severe OA patients may have resulted in a larger percentage of responders in both groups since these patients are more likely to benefit from their treatment for knee OA. The second study reported statistically significant differences on pain, function and stiffness (WOMAC questionnaire), and on PGA in favour of the intervention group [34]. A decrease of 38% in the pain scale in the intervention group was reported, compared to a 13% decrease in the control group. K&L grade IV was excluded but multiple series of intra-articular injections with HMW-HA were allowed, in contrast to 1 series of HMW-HA in our study. The effectiveness results of our study are in line with the results of both aforementioned studies. Including our study, the results of the 3 studies showed that the primary effectiveness outcome parameters improve at least 20% when HMW-HA is added to the usual care treatment.

Intra-articular injections with HMW-HA are frequently accompanied by transient pain or swelling of the knee. The procedure itself also includes a risk of inducing septic arthritis [16, 17]. At 6 weeks, a statistically significant difference of subjects receiving HMW-HA in our study reported flares or flare-like symptoms of the study knee compared to the control group (35% vs 10%, *p*= > 0.001) in the control group. No septic arthritis occurred. These results on local adverse events (AE) are similar compared to other studies [34, 35]. In our opinion the reduction of knee pain and the improvement of function outweigh the increase of transient flare like symptoms.

The follow up of the VISK study was 52 weeks. Optimal pain decrease after administration of intra-articular HMW-HA is seen at about 3 months though [16, 18]. A shorter follow-up period, closer to the peak effectiveness, encloses the risk of underestimation of possible health effects. Effects on pain function and PGA can occur during a longer period than the peak effectiveness. A longer follow-up also allows for assessment of the course of these effects. To ensure that these matters were accounted for, the current follow-up period of 52 weeks was chosen.

This study has limitations that need to be addressed. The study design of the VISK study did not include a placebo group. Previous research showed that placebo effects in intra-articular HA studies are above average [36]. It is thus likely that part of the beneficial effect in the intervention group is explained by the placebo effect. There were 2 main reasons to opt for this specific study design without a placebo group. First, evidence from high quality studies in meta-analyses showed that HMW-HA is efficacious for knee OA [18–20]. The next logical step was to investigate the actual effectiveness of HMW-HA, thereby accepting the fact that part of the possible beneficial effects is probably explained by the placebo effect. Second, a study design in which the intervention (HMW-HA) is compared to the usual care treatment (and not to placebo) in an everyday clinical setting is required to be able to facilitate a parallel economic evaluation which was also part of the VISK study project [25–27].

The target population of our study can be described as secondary care patients with symptomatic and mild to moderate knee OA. We therefore included subjects with K&L grade I-III and a minimal VAS pain score of 2. Patients who were more likely to benefit from surgical therapy like TKA or osteotomy, or from rheumatologic treatment where excluded in this study (e.g. K&L grade IV, substantial varus/valgus deformation, inflammatory arthritis). We aimed to avoid measuring effects strongly related to other factors than the intervention itself (e.g. recent or planned knee surgery, daily steroid use) and to avoid possible harm due to the intervention (e.g. allergies, pregnancy). Applying these criteria may have consequences for the generalizability of the results. It is for example uncertain if the effectiveness results also extend to other patient groups who might benefit from HMW-HA treatment, like knee OA patients not fit for surgery who are in need of surgical therapy.

## Conclusion

We conclude that intra-articular injections with HMW-HA added to usual care is effective in patients in the working age. It results in more responders to therapy and improvement in pain, function and PGA.

## Additional file

Additional file 1: Adverse events per study group per time point. Displays the amount of subjects experiencing treatment related and non-treatment related adverse events and their nature. (DOCX 20 kb)

## Abbreviations

HA: Hyaluronic acid
HMW: High molecular weight
K&L: Kellgren & Lawrence
KOOS: Knee injury and Osteoarthritis Outcome Score
NRS: Numeric rating scale
NSAIDs: Non-steroidal anti-inflammatory drugs
OA: Osteoarthritis
PGA: Patients global assessment
RCT: Randomized controlled trials
TKA: Total knee arthroplasty
UC: Usual care
VISK study: VIScosupplementation for Knee osteoarthritis study
