# Supplementary material for: The effectiveness of high molecular weight hyaluronic acid for knee osteoarthritis in patients in the working age: a randomised controlled trial
Source: BMC Musculoskelet Disord. 2019 May 7;20:196. doi: 10.1186/s12891-019-2546-8 (PMC6503549; doi:10.1186/s12891-019-2546-8)
Supplement: Supplementary file 1 — Adverse events per study group per time point. Displays the amount of subjects experiencing treatment related and non-treatment related adverse events and their nature. (DOCX 20 kb) [file 12891_2019_2546_MOESM1_ESM.docx]

Appendix 2 adverse events

| Patients experiencing knee treatment related adverse events (n=156) | | | | | | | | | | | |
| --- | --- | --- | --- | --- | --- | --- | --- | --- | --- | --- | --- |
|  |  | 6 | (n) | 13 | (n) | 26 | (n) | 39 | (n) | 52 | (n) |
|  | | intervention group (n=77) | | | | | | | | | |
| flare knee | | 36% | (27) | 8% | (6) | 8% | (6) | 8% | (6) | 6% | (5) |
| gastro-intestinal complaints | | 7% | (5) | 3% | (2) | 5% | (4) | 5% | (6) | 4% | (4) |
| other | | 11% | (8) | 8% | (8) | 5% | (5) | 4% | (3) | 4% | (3) |
| total | | 45% | (40) | 15% | (16) | 16% | (15) | 13% | (15) | 12% | (12) |
|  | | control group (n=79) | | | | | | | | | |
| flare knee | | 10% | (7) | 16% | (11) | 8% | (6) | 7% | (5) | 11% | (8) |
| gastro-intestinal complaints | | 6% | (7) | 12% | (11) | 3% | (3) | 4% | (3) | 3% | (3) |
| other | | 10% | (9) | 9% | (6) | 7% | (5) | 1% | (1) | 16% | (12) |
| total | | 18% | (23) | 27% | (28) | 15% | (14) | 11% | (9) | 20% | (23) |

| Patients experiencing other adverse events (n=156) | | | |
| --- | --- | --- | --- |
| intervention (n=77) | n | control group (n=79) | n |
| Removal of staple from tibia | 1 | Gout | 1 |
| Radius fracture | 1 | Spondylolisthesis | 1 |
| Fibroadenoma | 1 | Removal of seborrheic verruca | 1 |
| Abducens nerve paresis | 1 | Partial parotidectomy due to atypical Whartin tumor | 1 |
| Peroneal tendon ganglion | 1 | Dermatological flebectomy | 1 |
| Ribfracture | 1 | Actinic keratosis | 1 |
| Neurofibromatosis | 1 |  |  |
